# Molecular assessment of splicing variants in a cohort of patients with inborn errors of immunity: methodological approach and interpretation remarks

**DOI:** 10.3389/fimmu.2024.1499415

**Published:** 2025-01-29

**Authors:** Laura Miguel Berenguel, Carla Gianelli, Elisabet Matas Pérez, Teresa del Rosal, Ana Méndez Echevarría, Ángel Robles Marhuenda, Marta Feito Rodríguez, Maria Teresa Caballero Molina, Lorena Magallares García, Brenda Sánchez Garrido, Samantha Hita Díaz, Luis Allende Martínez, Pilar Nozal Aranda, Carmen Cámara Hijón, Eduardo López Granados, Rebeca Rodríguez Pena, María Bravo García-Morato

**Affiliations:** ^1^ Department of Immunology, La Paz University Hospital, Madrid, Spain; ^2^ Center for Biomedical Network Research on Rare Diseases (CIBERER U767), Madrid, Spain; ^3^ Lymphocyte Pathophysiology in Immunodeficiencies Group, La Paz Institute of Biomedical Research, Madrid, Spain; ^4^ Department of Pediatric Infectious Diseases, La Paz University Hospital, Madrid, Spain; ^5^ Department of Internal Medicine, La Paz University Hospital, Madrid, Spain; ^6^ Department of Dermatology, La Paz University Hospital, Madrid, Spain; ^7^ Department of Allergy, La Paz University Hospital, Madrid, Spain; ^8^ Center for Biomedical Network Research on Rare Diseases (CIBERER U754), Madrid, Spain; ^9^ Department of Pediatric Gastroenterology, La Paz University Hospital, Madrid, Spain; ^10^ Immunology Department, 12 de Octubre University Hospital, Madrid, Spain; ^11^ Research Institute Hospital 12 Octubre (I+12), Madrid, Spain; ^12^ Complement Alterations in Human Pathology Group, La Paz Institute of Biomedical Research, Madrid, Spain

**Keywords:** splicing mutations, reverse-transcription PCR (RT-PCR), inborn errors of immunity, genetic validation algorithm, canonical sites of splicing, deep-intronic variants

## Abstract

**Background:**

Splicing is the molecular mechanism to produce mature messenger RNA (mRNA) before its translation into protein. It is estimated that 50% of disease-causing mutations disrupt splicing, mostly of them affecting canonical positions. However, variants occurring in coding regions or deep-intronic variants can also affect splicing. In these cases, interpretation of the results may be challenging and molecular validation is required.

**Methods:**

The study includes 23 patients with splicing variants out of a cohort of 187 patients diagnosed with inborn errors of immunity (IEI). Clinical features and immunophenotypes are shown. Reverse transcription-polymerase chain reaction (RT-PCR) is the molecular assay employed for pathogenicity validation.

**Results:**

We detected 23 patients of 20 pedigrees with splicing variants in IEI genes, which constitutes the 12.3% of our cohort. In total, 21 splicing variants were analyzed, 10 of which had previously been reported in the literature and 11 novel ones. Among the 23 patients, 16 showed variants at canonical splice sites. Molecular validation was required only in the cases of genes of uncertain significance (GUS), high homology pseudogenes or incompatible clinical phenotype. Seven patients showed variants outside canonical positions. All of them needed molecular validation, with the exception of two patients, whose variants had previously been well characterized in the medical literature.

**Conclusion:**

This study shows the proportion of splicing variants in a cohort of IEI patients, providing their clinical phenotypic characteristics and the methodology used to validate the splicing defects. Based on the results, an algorithm is proposed to clarify when a splicing variant should be validated by complementary methodology and when, by contrast, it can be directly considered disease causing.

## 1 Introduction

Splicing is the molecular mechanism to produce mature messenger RNA (mRNA) before its translation into protein. Intronic DNA resides within genes and needs to be removed from primary transcripts, which results in mature mRNA containing only coding sequences (1). For this purpose, the spliceosome, that recognizes exon-intron junctions, catalyzes the removal of introns and mediates exons joining (2). In most cases (98.7%), the exon-intron boundary sequences contain GT and AG motifs at the 5´and 3´ends of the intron and are considered the canonical donor and acceptor sites of the splicing. These dinucleotides are evolutionary conserved and are essential for the correct splicing process and proper protein synthesis (3).

It is estimated that 50% of disease-causing mutations affects splicing (4). Most of the pathogenic splicing mutations are single nucleotide substitutions in the donor or acceptor splice sites that involve the +1/+2 or -1/-2 positions. This leads to an improper gene transcription into mRNA, which usually results in exon skipping or intron retention (2). In addition, variants occurring in coding regions or deep-intronic variants can also disrupt splicing by either activating a cryptic splice site or by interfering with splicing regulatory elements (5). In these cases, the use of *in silico* splicing predictors are imprecise and the consequences at the mRNA level may be more heterogeneous (6). Absence of functional assays or specific validation methods has limited the correct classification of these variants and, therefore, the extent of pathogenic splicing variants has probably been underestimated (7).

To demonstrate the pathogenic effect of an splicing mutation, methodology such as reverse transcription-polymerase chain reaction (RT-PCR) or the mini-gene assay can be performed. The first one is based on the retro-transcription of the mRNA pool of a proband into complementary DNA (cDNA) and the amplification of a target gene. The cDNA sequence can be isolated and sequenced in subsequent steps, demonstrating the presence or absence of an abnormal mRNA product derived from an aberrant splicing (8). Mini-gene assay, by contrast, uses circular plasmids to clone the exon and the flanking intronic sequence both in the wild type and the aberrant form and, after transfection into cultured cells, transcripts are compared (9). RT-PCR can be also useful to analyse genes with pseudogenes as it has been reported that 90% of pseudogenes are not transcribed (10).

This study shows the proportion of splicing variants in a cohort of 187 patients with confirmed genetic diagnosis of inborn errors of immunity (IEI), providing their clinical phenotypic characteristics and the methodology used to validate the splicing defects. Based on the results, an algorithm is proposed to clarify when a splicing variant should be validated by complementary methodology and when, by contrast, it can be directly considered disease causing.

## 2 Materials and methods

### 2.1 Patients

The study includes 23 patients (20 pedigrees) with splicing variants out of a cohort of 187 patients (157 pedigrees) diagnosed with IEI attended at La Paz University Hospital in Madrid, Spain, between 2014 and 2024 The whole cohort included autoinflammatory diseases (26 patients of 22 pedigrees) and hereditary angioedema (37 patients of 31 pedigrees), among others. Patients underwent an anamnesis and an immunological analysis that led to a suspicion of IEI. In 10/23 patients, these variants were detected by Sanger sequencing while in the rest of the patients a next generation sequencing (NGS)-customized panel or whole exome sequencing (WES) was performed. For NGS data analysis, only the variants that appeared exclusively in the patient and in no other sample with an allelic frequency of less than 1% were considered.

### 2.2 DNA extraction

The extraction of genomic DNA from whole blood was performed with a Chemagic 360/Chemagen Magtration system 8Lx (PerkinElmer, Waltham).

### 2.3 Sanger sequencing

DNA was amplified with self-designed primers using Master Mix (Promega, Madison). PCR products were run on 2% agarose gels and purified using the *ExS-Pure* system (NimaGen, Nijmegen). Sanger sequencing was performed using the BrightDye terminator sequencing kit, (NimaGen, Nijmegen).

### 2.4 NGS sequencing

DNA of some patients was subjected to mutation screening using a customized NGS gene panel containing all genes associated with IEI at the time, which was periodically updated in accordance with the evolving IEI classification by the International Union of Immunological Societies (IUIS). This panel was designed with NimbleDesign software (https://design.nimblegen.com). For each sample, paired-end libraries were created with the help of KAPA HTP Library Preparation Kit for Illumina platforms, SeqCap EZ Library SR and NEXTflex-96 Pre-Capture Combo Kit for indexing. Sequencing was conducted on a NovaSeq system (Illumina, San Diego) according to the standard operating protocol.

For WES, Nextera Rapid Capture Exome system (Illumina, San Diego) was used. Libraries were created and sequenced on a NextSeq (Illumina) following the manufacturer’s standard protocol.

### 2.5 RT-PCR

Total RNA was isolated using the miRNeasy Micro Kit (Qiagen, Hilden) after isolating mononuclear cells through a Ficoll-Hypaque gradient (GE Healthcare, Chicago). The purified mononuclear cells were resuspended in RPMI 1640 complete medium (Gibco, Waltham) supplemented with 2 mM L-glutamine (Life Technologies, Carlsbad), 100 U/ml penicillin (Invitrogen, Carlsbad), 100 μg/ml streptomycin (Invitrogen, Carlsbad), and 10% fetal bovine serum (Invitrogen, Carlsbad). cDNA was synthesised using the High-Capacity RNA to cDNA Kit (Applied Biosystems, Foster City). The strategy to perform RT-PCR was based on the nested PCR amplification of the cDNA corresponding to the full-length messenger using a forward primer that hybridizes in the 5’ UTR and a reverse primer that hybridizes in the 3’ UTR with Master Mix (Promega, Wisconsin). Primer sequences are provided in **Supplementary Table 1**. Subsequently, PCR products were run on 2% agarose gels and purified with the QiAquick kit (Qiagen, Hilden). Then, smaller fragments were re-amplified using self-designed primers complementary to exon-exon junctions specific to the transcript of interest. Sanger sequencing was performed using the BrightDye terminator sequencing kit (Nimagen, Nijmegen).

RT-PCR of the *CTLA4* and *IRF4* genes required a pre-RNA extraction step involving cellular activation with phorbol 12-myristate 13-acetate (PMA) 20 ng/ml and ionomycin 1 μg/ml (both Sigma-Aldrich, San Luis) to induce the expression of the gene’s mRNA, as they are not constitutively expressed.

### 2.6 Lymphocyte proliferation assays

Heparinized whole blood from the patients were cultured with mitogens (PHA [Life Technologies, Carlsbad], concanavalin A [Cayman Chemical, Ann Arbor], pokeweed mitogen [Sigma-Aldrich, San Luis], and OKT3 [BD Biosciences, Madrid]) or antigen (candidin; Calbiochem, San Diego) and analyzed by using tritiated thymidine uptake (Amersham Biosciences, Piscataway) after 3 (mitogens) or 6 (antigen) days.

### 2.7 Protein expression by flow cytometry

Samples were treated with the systems outlined in **Table 1**.

**Table 1:** Reagents and monoclonal antibodies used for the detection of intracellular proteins by flow cytometry.

*Protein*	*Cellular activation*	*Fixing/Permeabilizing*	*Primary antibody*	*Secondary antibody*	*Gating antibodies*
*BTK*	–	Formaldehyde, Triton-X, Methanol	Monoclonal antibody anti-BTK Clon: D3H5, rabbit (Cell signaling, Danvers)	Rabbit antibody anti-IgG (H+L), fragment F(ab´)2-AF488 (Cell signalling, Danvers)	CD14-PC5.5 CD19-PC7 (all from Beckman Coulter, Brea)
*SAP*	–	Formaldehyde, Triton-X, Methanol	Monoclonal antibody anti-SAP Clon: 1C9, mouse (Abnova, Taipei)	Mouse antibody anti-IgG (H+L), fragment F(ab´)2- AF488 (BD Biosciences, Madrid)	CD3-ECD CD8-AF700 CD4-AF750 CD16-APC CD56-APC CD19-PC7 (all from Beckman Coulter, Brea)
*IRF4*	PMA 60 ng/mL and ionomycin 1 μg/ml (Sigma-Aldrich, San Luis) Anti-human CD3/CD28 (Life Technologies, Carlsbad)	FoxP3 fixation/permeabilization working solution (eBioscience, San Diego)	Monoclonal antibody anti-human IRF4-PE Clon: 3E4, rat (eBioscience, San Diego)	–	CD3-FITC, CD27-BV421 CD19–PE-Cy7 (all from BD Biosciences, Madrid)
*CTLA4*	OKT3 1 mg/mL (BD Biosciences, Madrid)	FoxP3 fixation/permeabilization working solution (eBioscience, San Diego)	Monoclonal antibody anti- human CD152-PE Clon: BNI3, mouse (Beckman Coulter, Brea)	–	APC-AF750-CD4 APC-AF700-CD8 PCY7-CD25 FITC-CD127 FOXP3-AF647 (all from Beckman Coulter, Brea)

For the assays performed with formaldehyde, Triton X, and methanol, 100 μl of total blood were treated with 65 μl of 10% formaldehyde, 1 ml of 0.1% Triton X-100 and 1 ml of 50% methanol.

For the assays performed with the Transcription Factor Buffer kit (BD Biosciences, Madrid), PBMCs isolated by Ficoll-Hypaque (GE Healthcare, Fairfield) were treated according to the manufacturer’s instructions.

### 2.8 Functional assays

#### 2.8.1 NK and CD8 degranulation assay

For degranulation assays to quantify cell-surface CD107a expression, 10^5^ resting PBMCs were washed twice in PBS and added to 2x10^5^ K562 cells in 200 μl of complete medium consisting of RPMI 1640 (Gibco, Waltham) supplemented with 2 mM L-glutamine (Life Technologies, Carlsbad), 100 U/ml penicillin (Invitrogen, Carlsbad), 100 μg/ml streptomycin (Invitrogen, Carlsbad), and 10% fetal bovine serum (Invitrogen, Carlsbad). Cells were centrifuged (600 rpm for 3 minutes) and incubated (3 hours at 37°C in a 5% CO_2_ atmosphere). Then, cells were stained with CD3-ECD, CD56-APC and PB-CD107a monoclonal antibodies (all from Beckman Coulter, Brea) and analyzed by flow cytometry.

#### 2.8.2 C1 inhibitor functional assay

C1INH functionality in plasma was quantified by the Berichrom^®^ chromogenic assay (Siemens Healthcare Diagnostics, Eschborn).

## 3 Results

**Figure 1:**
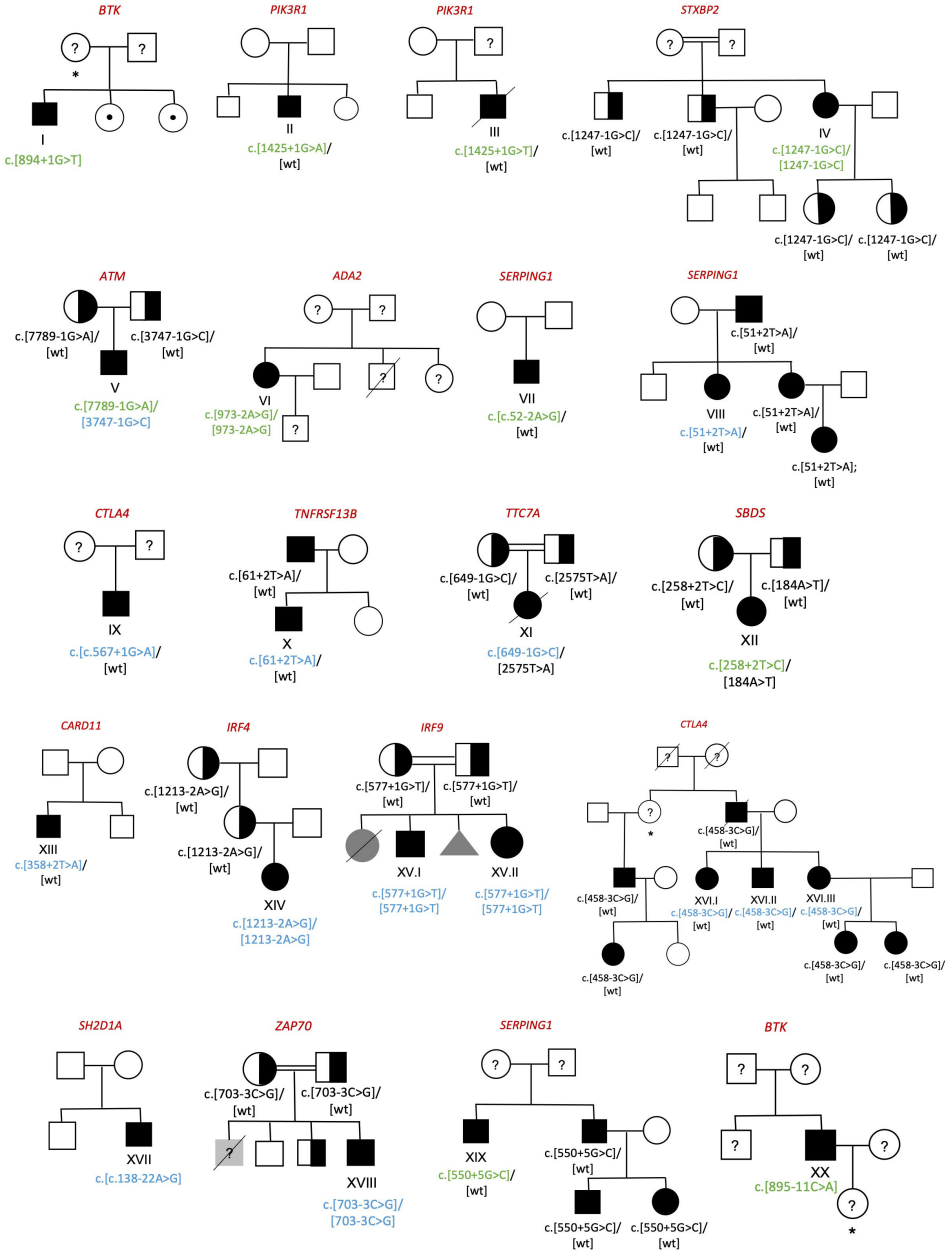
Family pedigrees of diagnosed individuals. Circle=woman; Box=man; Triangle=abortion; Black symbol=affected; White symbol=unaffected; Grey symbol= individuals with clinical phenotype without genetic study; Half-filled symbol=healthy carrier; Symbol with center dot=healthy carrier of an X-linked disease; Crossed out symbol=deceased; Question marker=unstudied individual; *=obligate carrier. Previously described splicing variants are shown in green whereas the novel ones are shown in blue.

We detected 23 patients of 20 pedigrees with splicing variants in IEI genes (**Figure 1**), which constitutes the 12.3% of our cohort of 187 patients. In the patients who underwent NGS, either through gene panel or WES, a comprehensive analysis of all variant types was performed, and no additional clinically relevant variants were identified. In total, 21 splicing variants were analyzed, 10 of which had previously been reported in the literature and 11 novel ones.

### 3.1 Variants at canonical splice sites

Among the 23 patients, 16 showed variants at canonical splice sites (**Table 2**). Attending to the American College of Medical Genetics and Genomics (ACMG) guidelines for interpretation of variant pathogenicity (11) and considering the phenotypes correlation in all cases, most of the variants were classified as disease causing (pathogenic, likely pathogenic) without the necessity of molecular validation. However, for patients XII, XIII, XIV and XV.I validation by RT-PCR was necessary to conclude pathogenicity. Additionally, in patients I, IV, VII, VIII and IX protein expression was performed to support the molecular defect, although it was not indispensable.

**Table 2:** Patients with splicing variants at canonical splice sites. In compound heterozygous patients, missense variants were classified as likely pathogenic/pathogenic according to ACMG criteria.

Patient ID	Age at diagnosis Sex	Variants Method	Origin Cigosity	IEI Inheritance pattern	RT-PCR (reasons) Abnormalities on mRNA Predicted protein	Functional assay	Immunological and clinical features	Previously described variant (references)
I	35y Male	*BTK*: NM_000061.3: c.894+1G>T Sanger sequencing	(Probably) Maternal The mutation was present in both sisters Hemizygous	BTK deficiency, X-linked agammaglobulinemia (XLA) X-linked	NO	Impaired BTK expression by flow cytometry	Lymphopenia (890/µL; RV: 1200-4100*). Absent B cells. Agammaglobulinemia. Normal proliferation assays. No relevant infections.	YES (34–36)
II	10y Male	*PIK3R1*: NM_181523.3: c.1425+1G>A Sanger sequencing	*De novo* Heterozygous	APDS2 and SHORT syndrome Autosomal dominant	NO		Normal total lymphocytes counts. Low percentages of CD4+ (16%; RV: 28.4-44.4***) and CD19 (3%; RV: 4-33*). IgA deficiency (<7 mg/dL; RV: 45-236**) with normal IgG and IgM**. Moderately reduced proliferation assays. Poor weight gain, mitral stenosis, pulmonary hypertension, mild motor and speech delay, recurrent infections, hearing loss, lymphadenopathies, dysmorphic features.	YES (37–42)
III	23m Male	*PIK3R1*: NM_181523.3: c.1425+1G>T Sanger sequencing	Unknown Heterozygous	APDS2 and SHORT syndrome Autosomal dominant	NO		Low %CD19 (6%; RV: 8-45*). Absent IgA with low IgG (36 mg/dL; RV: 345-1213**) and normal IgM. Moderately reduced proliferation assays. Poor growth and weight gain, facial dysmorphism, dysplastic mitral valve and aortic valve insufficiency, delayed teeth eruption, autoimmune hypothyroidism, atrophic gastritis, recurrent infections, hearing loss.	YES (40, 41)
IV	61y Female	*STXBP2*: NM_006949.4: c.1247-1G>C Customized NGS gene panel	Unknown Homozygous	STXBP2/Munc 18-2 deficiency (FHL5) Autosomal dominant or recessive	NO	Impaired CD107a degranulation assay	Normal total lymphocyte counts. Absent NK cells. Normal IgG, IgA and IgM. Fever, hepatosplenomegaly, adenopathies.	YES (43–49)
V	3y Male	*ATM*: NM_000051.4: c.7789-1G>A *ATM*: NM_000051.4: c.3747-1G>C Sanger sequencing	Maternal Paternal Compound heterozygous	Ataxia-telangiectasia Autosomal recessive	NO		Lymphopenia (630/µL; RV: 1400-5500*). Low CD4+ (25%; RV: 28.1-43.2***) and low %CD45RA+CD31+T cells (3.9%; RV: 53.2-67.5***). Normal IgG, IgA and IgM. Reduced proliferation assays. Ataxia, telangiectasia of the eyes.	YES (50) NO
VI	53y Female	*ADA2*: NM_001282225.2: c.973-2A>G Sanger sequencing	Unknown Homozygous	ADA2 deficiency Autosomal recessive	NO		Lymphopenia (670/µL; RV: 1200-4100*). Low switched memory B cells (0.97%; RV: 3-46*). Low IgG (363 mg/dL; RV: 639-1349**), IgA (6 mg/dL; RV: 70-312**) and IgM (50 mg/dL; RV: 56-352**). Normal proliferation assays. Recurrent respiratory infections, *Giardia* and *H. pylori* infections, bronchiectasis, psoriasis, acute promyelocytic leukemia.	YES (51)
VII	31y Male	*SERPING1*: NM_000062.3: c.52-2A>G Sanger sequencing	*De novo* Heterozygous	C1 inhibitor deficiency Autosomal dominant	NO	Impaired C1INH functional assay (12.05 % compared to a healthy donor)	Low serum C4 (3.40 mg/dL; RV: 15-45**) and C1INH (<7.21 mg/dL; RV: 16-33 mg/dL). Several episodes of facial edema after surgeries. Recurrent episodes of local edema after minor trauma.	YES (52)
VIII	77y Female	*SERPING1*: NM_000062.3: c.51+2T>A Sanger sequencing	Paternal Heterozygous	C1 inhibitor deficiency Autosomal dominant	NO	Impaired C1INH functional assay (25.25% compared to a healthy donor)	Low serum C4 (6.56 mg/dL; RV: 15-45**) and C1INH (3.89 mg/Dl; RV: 16-33 mg/dL). Recurrent episodes of edema in different locations, including respiratory tract. Frequent episodes of abdominal pain, nausea and vomiting.	NO
IX	39y Male	*CTLA4*: NM_005214.5: c.567+1G>A Customized NGS gene panel	Unknown Heterozygous	CTLA4 haploinsufficiency Autosomal dominant	NO	Impaired CTLA4 expression by flow cytometry	Lymphopenia (610/µl; RV: 1200-4100*). Low switched memory B cells (0.30%; RV: 3-46*) and CD21 low expansion (48.70%; RV: 2-14*). Absent IgA and IgM; Pre-replacement therapy IgG levels are not available. Reduced proliferation assays. Recurrent respiratory low infections, adenopathies, splenomegaly, atrophic gastritis.	NO
X	18y Male	*TNFRSF13B*: NM_012452: c.61+2T>A Customized NGS gene panel	Paternal Heterozygous	TACI deficiency (condition of predisposition to common variable immunodeficiency syndrome) Autosomal dominant or recessive	NO		Normal total lymphocyte counts and TBNK subpopulations. Low serum IgG (379 mg/dL; RV: 639-1349**) and IgA (16 mg/dL; RV: 70-312**) with normal IgM. Normal proliferation assays. Autoimmune hepatitis.	NO
XI	1y Female	*TTC7A*: NM_020458.4: c.649-1G>C *TTC7A*: NM_020458.4: c.2575T>A Sanger sequencing	Maternal Paternal Compound heterozygous	Immunodeficiency with multiple intestinal atresias Autosomal recessive	NO		Lymphopenia (700/µL; RV: 3200-12300*). Low IgG (332 mg/dL; RV: 345-213**), IgA (<8 mg/dL; RV: 14-106*) and IgM (<5 mg/dL; RV: 43-173**). Reduced proliferation assays. Multiple intestinal atresias, hepatopathy, coagulopathy, *Klebsiella pneumonia*e sepsis, portal hypertension with gastrointestinal bleeding.	NO
XII	2y Female	*SBDS*: NM_016038.4: c.258+2T>C *SBDS:* NM_016038.4: c.184A>T Sanger sequencing	Maternal Paternal Compound heterozygous	Shwachman-Diamond syndrome Autosomal recessive	YES (High homology pseudogene) Deletion of 8 nucleotides within exon 2 (GTAAGCAG) p.(Cys84TyrfsTer4)		Neutropenia (1000/µL; RV: 1500-8500****). Normal total lymphocyte counts, immunoglobulins, TBNK subpopulation and proliferation assays. Exocrine pancreatic insufficiency, bone dysplasia, persistent eccema, bronquiolitis, poor weight gain.	YES (53)
XIII	2y Male	*CARD11*: NM_032415.7: c.358+2T>A WES	*De novo* Heterozygous	CARD11 deficiency Autosomal dominant or recessive	YES (Patient´s phenotype consistent with the one described for dominant negative variants) Exon 4 skipping p.(Gly74_Val119del)		Intermittent neutropenia (810/ µL; RV: 1500-8500****). Normal lymphocyte counts. Expansion of B transitional cells (47.74%; RV: 0.7-24*). Absent IgA with normal IgG and IgM. Elevated IgE (96 kU/L). Reduced proliferation assays. Decreased specific anti-polisacaridic antibodies after vaccination. Recurrent respiratory infections, sepsis adenopathies, diarrheas, oral ulcers, atopic dermatitis, failure to thrive.	NO
XIV	2y Female	*IRF4*: NM_002460.4: c.1213-2A>G WES	Maternal (uniparental disomy) Homozygous	IRF4 haploinsufficiency Autosomal recessive	YES (GUS) Addition of 1 intronic nucleotide between exons 8 and 9 p. (Val405GlyfsTer127)		Hypereosinophilia (26%; RV: 0.5-5****). Normal lymphocyte counts. Low B cells (6%; RV: 8-45*) and absent memory B cells. Agammaglobulinemia. Reduced proliferation assays (PHA). Intrauterine growth retardation, severe dermatitis, failure to thrive, long periods of fever without focus, bronchopneumonia, muguet and diarrhea.	NO
XV.I	11y Male	*IRF9*: NM_006084.5: c.577+1G>T Customized NGS gene panel	Maternal and paternal Homozygous	IRF9 deficiency Autosomal recessive	YES (GUS) Exon 5 skipping p. (Glu166LeufsTer80)		Lymphopenia (70/µL; RV: 1400-4200*). Low CD4+ lymphocytes (13%; RV: 28.4-44.4***). Low serum IgG (252 mg/dL; RV: 639-1349**) IgA (37 mg/dL; RV: 70-312**) and IgM (24 mg/dL; RV: 56-352**). Reduced proliferation assays. Recurrent severe viral infections.	NO
XV.II	1y Female	*IRF9*: NM_006084.5: c.577+1G>T Sanger sequencing	Maternal and paternal Homozygous	IRF9 deficiency Autosomal recessive	Carried out in patient XV.I (GUS) Exon 5 skipping p. (Glu166LeufsTer80)		Substitutive treatment and prophylaxis since birth. No infections and no relevant immunological findings.	NO

Patients II and III, XIV and XV.I have previously been reported by Bravo García-Morato et al. (19, 20, 54). Y, years old; M, months old; GUS, Gene of Uncertain Significance. Reference values (RV) obtained from *Schatorjé EJ et al. *Scand J Immunol.* 2011 Nov;74(5):502–10; **Jolliff CR et al. *Clin Chem.* 1982 Jan;28(1):126–8; ***van Gent R et al. *Clin Immunol.* 2009 Oct;133(1):95–107; ****Herklotz R et al. *Ther Umsch.* 2006; 63(1): 5-24.

**Figure 2:**
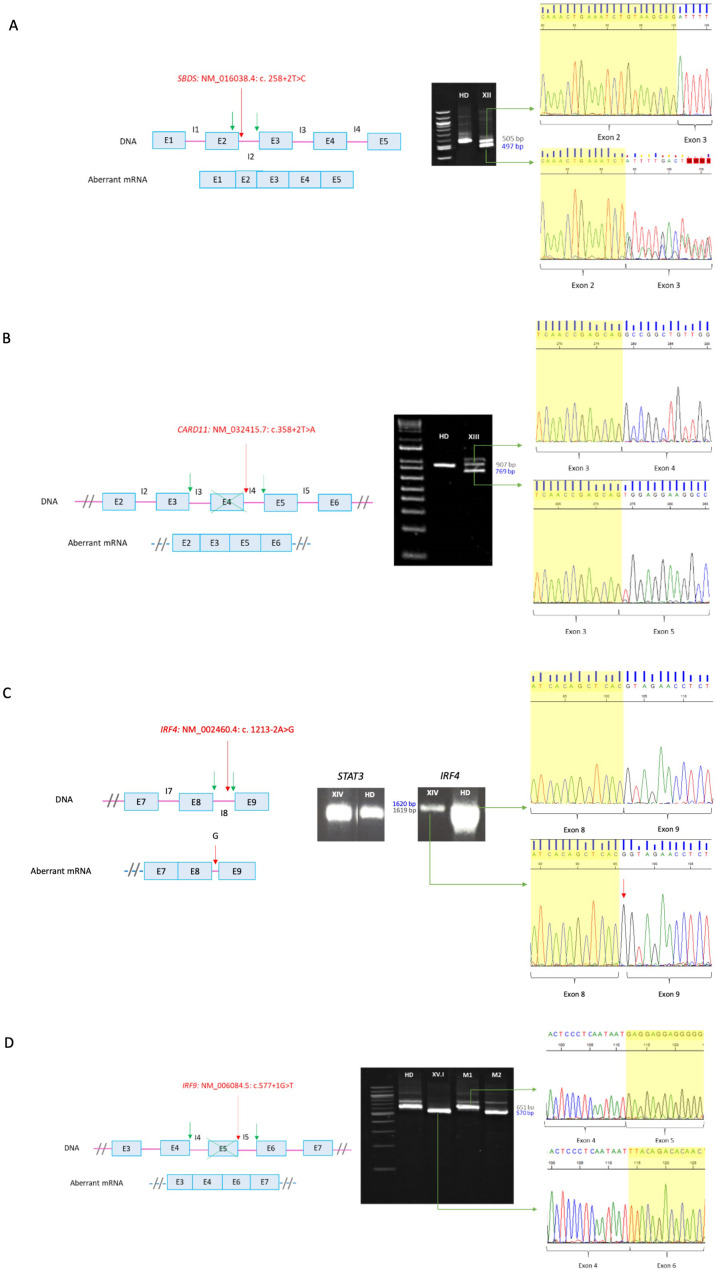
RT-PCR of canonical splicing defects. Base pairs corresponding to fragments of the wild type (wt) mRNA are shown in grey and those of the aberrant ones in blue. Red arrows indicate splicing variants positions and green ones point the new splicing sites. E, exon; I, intron; HD, healthy donor; P, proband; M, mother. **(A)** *SBDS*: NM_016038.4: c. 258+2T>C. Loss of 8 bp within exon 2 on aberrant mRNA. A double sequence is observed due to a contamination of the aberrant mRNA band with the wild-type one. **(B)** *CARD11*: NM_032415.7: c.358+2T>A. In-frame exon 4 skipping on aberrant mRNA. The upper band corresponds to the combination of the wild-type mRNA with the aberrant one. **(C)** *IRF4*: NM_002460.4: c. 1213-2A>G. Introduction of an intronic G into the mRNA sequence. The aberrant mRNA is not distinguishable in size from the wild-type one by electrophoresis. **(D)** *IRF9*: NM_006084.5: c.577+1G>T. Exon 5 skipping on aberrant mRNA. M1 and M2 correspond to positions NM006084.5: c.350_999 of the carrier mother belonging to the wt and the aberrant mRNA, respectively. Electrophoresis **(A, B, D)** correspond to the reamplification of two fragments representing the wild type and the aberrant mRNA, obtained from the amplification of both full-length mRNA, whereas electrophoresis C corresponds to the amplification of the full-lenght mRNA, from 5´UTR to 3´UTR.

For patient XII (*SBDS*: NM_016038.4: c. 258 + 2T>C), RT-PCR was required since *SBDS* gene has a high homology pseudogene which is not transcribed into mRNA. Thus RT-PCR in this case allowed us to confirm that the variant was in the gene sequence and was disease causing. Analyses of the RT-PCR product showed an additional smaller band in the patient sample, which was absent in the healthy donor (**Figure 2A**). Sanger sequencing showed an aberrant mRNA that loosed 8 nucleotides within exon 2, leading to a frameshift and a premature stop codon [p.(Cys84TyrfsTer4)].

For patient XIII (*CARD11*: NM_032415.7: c.358 + 2T>A), we carried out an RT-PCR since his phenotype was consistent with the one described for dominant negative variants (CADINS). The majority of the variants that lead to this mechanism are missense, whereas heterozygous splicing variants are generally associated with loss of function mechanisms leading to haploinsufficiency. In addition, haploinsufficiency is not associated with pathology for the *CARD11* gene, so validation of the splicing variant was indicated. RT-PCR in the patient (**Figure 2B**) yielded two main cDNA fragments. The sequence of the smaller fragment showed an in-frame exon 4 skipping. The resulting protein theoretically has a deletion of residues 74 to 119, encompassing the end of the CARD domain and the beginning of the LATCH domain, where most missense variants with a dominant-negative effect have been identified (12). Thus, we hypothesized that the loss of residues 74-119 may lead to the synthesis of an abnormal protein that can exert a dominant negative effect, instead of avoiding protein’s expression.

The variant detected in patient XIV (*IRF4*: NM_002460.4: c. 1213-2A>G) was in a gene of uncertain significant (GUS) at the time the study was conducted. Since this gene had not been previously associated with human pathology, it was necessary to describe the mechanism for the disease. For this purpose, analyzing mRNA is an important step, although other experiments are required. A unique band was identified both in the patient and in the healthy donor (**Figure 2C**). However, the amount of cDNA in the patient was found to be significantly lower compared to the healthy donor. Signal transducer and activator of transcription 3 (*STAT3*) was amplified in parallel as a loading control, which allowed us to hypothesize that nonsense-mediated decay (NMD) was occurring. After sequencing, the band of the patient showed the introduction of an intronic G into the mRNA sequence between exons 8 and 9 causing a frameshift and an extension of 80 amino acids [p.(Val405GlyfsTer127)].

*IRF9* was also a GUS when the study was carried out, so RT-PCR was also required for patient XV.I (*IRF9*: NM_006084.5: c.577 + 1G>T). Electrophoresis results showed a unique band in the healthy donor and a shorter band in the patient, whereas her mother (healthy carrier of the mutation) presented both of them (**Figure 2D**). Aberrant mRNA in the patient showed an exon 5 skipping leading to a frameshift and a premature stop codon [p.(Glu166LeufsTer80)].

### 3.2 Variants outside the canonical splice sites

Seven patients had variants outside the canonical positions (**Table 3**). In most cases, molecular validation was required to reach a conclusive IEI diagnosis as pathogenicity could not be confirmed attending to ACMG criteria.

**Table 3:** Patients with splicing variants located outside canonical positions.

Patient ID	Age at diagnosis Sex	Variants Method	Origin Cigosity	IEI	RT-PCR Abnormalities on mRNA abnormal protein	Functional assay	Immunological and clinical features	Previously described (references)
XVI.I	45y Female	*CTLA4:* NM_005214.5: c.458-3C>G Sanger sequencing	Paternal Heterozygous	CTLA4 haploinsufficiency (ALPS-V) Autosomal dominant	YES Exon 3 skipping p.(Asp153AlafsTer23)	Impaired CTLA4 expression by flow cytometry	Thrombocytopenia (64000/µL; RV: 150000-370000***). Normal lymphocyte counts and TBNK subpopulations. Low IgG (382 mg/dl; RV: 639-1349**), IgA (29 mg/dl; RV: 70-312**) and IgM (20 mg/dl; RV: 56-352). Normal proliferation assays. Atopic dermatitis, adenopathies, recurrent respiratory infections, diarrhoeas.	NO
XVI.II	42y Male	*CTLA4:* NM_005214.5: c.458-3C>G Customized NGS gene panel	Paternal Heterozygous	CTLA4 haploinsufficiency (ALPS-V) Autosomal dominant	Carried out in patient XVI.I Exon 3 skipping p.(Asp153AlafsTer23)		Normal lymphocyte counts and TBNK subpopulations. Low IgM (26 mg/dl; RV: 56-352**) with normal IgG and IgA. Normal proliferation assays. Atopic dermatitis, alopecia areata.	NO
XVI.III	40y Female	*CTLA4:* NM_005214.5: c.458-3C>G Sanger sequencing	Paternal Heterozygous	CTLA4 haploinsufficiency (ALPS-V) Autosomal dominant	Carried out in patient XVI.I Exon 3 skipping p.(Asp153AlafsTer23)		Lymphopenia (620/µL; RV: 1200-4100*). Normal TBNK subpopulations. Absent IgA with normal IgG and IgM. Normal proliferation assays. Atopic dermatitis.	NO
XVII	6y Male	*SH2D1A*: NM_002351.5: c.138-22A>G Sanger sequencing	*De novo* Hemizygous	SAP deficiency (XLP1) X-linked	YES Exon 2 skipping p.(Tyr47GlnfsTer13)	Impaired SAP expression by flow cytometry	Normal lymphocyte counts. Low switched memory B cells (2.35%; RV: 3-18*). Absent IgG and IgA, low IgM (14 mg/dL; RV: 48-207**). Detectable EBV viral loads. Recurrent respiratory infections.	NO
XVIII	2m Male	*ZAP70*: NM_001079: c.703-3C>G Customized NGS gene panel	Maternal and paternal Homozygous	ZAP-70 deficiency Autosomal recessive	YES Intron 5 retention p.(Leu235ValfsTer95)		Normal lymphocyte counts and TBNK subpopulations^∞^. Low IgG (279 mg/dl; RV: 608-1572**), IgA (10 mg/dl; 45-236**) and IgM (50 mg/dl; RV: 52-242**). Reduced proliferation assays. Recurrent infections, failure to thrive, hematopoietic stem cell transplantation at 9 months of age.	NO
XIX	71 Male	*SERPING1*: NM_000062.3: c.550+5G>C Sanger sequencing	Unknown Heterozygous	C1 inhibitor deficiency Autosomal dominant	NO	Impaired C1INH functional assay (31.64% compared to a healthy donor)	Low serum C4 (11.40 mg/dl; RV: 15-45**) and C1INH (6.90 mg/dL; RV: 16-33 mg/dL) Recurrent episodes of swelling of the hands since childhood and one episode of upper airway edema.	YES (11)
XX	30y Male	*BTK*: NM_000061.3: c.895-11C>A Sanger sequencing	Maternal Hemizygous	BTK deficiency, X-linked agammaglobulinemia (XLA) X-linked	NO		Absent B cells. Low serum IgG (358 mg/dL; RV: 639-1349**) and IgM (59 mg/dL; RV: 56-352**), with elevated oligoclonal IgA (497 mg/dL; RV: 70-312**). Decreased specific anti-polisacaridic antibodies after vaccination. Recurrent pneumonias, oral ulcers and odynophagia.	YES (12)

Y, years old; M, months old. ∞: neither lymphocytosis nor the characteristic defect of CD8 T cells associated with ZAP70 deficiency were observed, probably due to the early hematopoietic stem cell transplantation at two months of age. Reference values (RV) obtained from *Schatorjé EJ et al. *Scand J Immunol.* 2011 Nov;74(5):502–10; **Jolliff CR et al. *Clin Chem.* 1982 Jan;28(1):126–8; *** Herklotz R et al. *Ther Umsch.* 2006; 63(1): 5-24.

**Figure 3:**
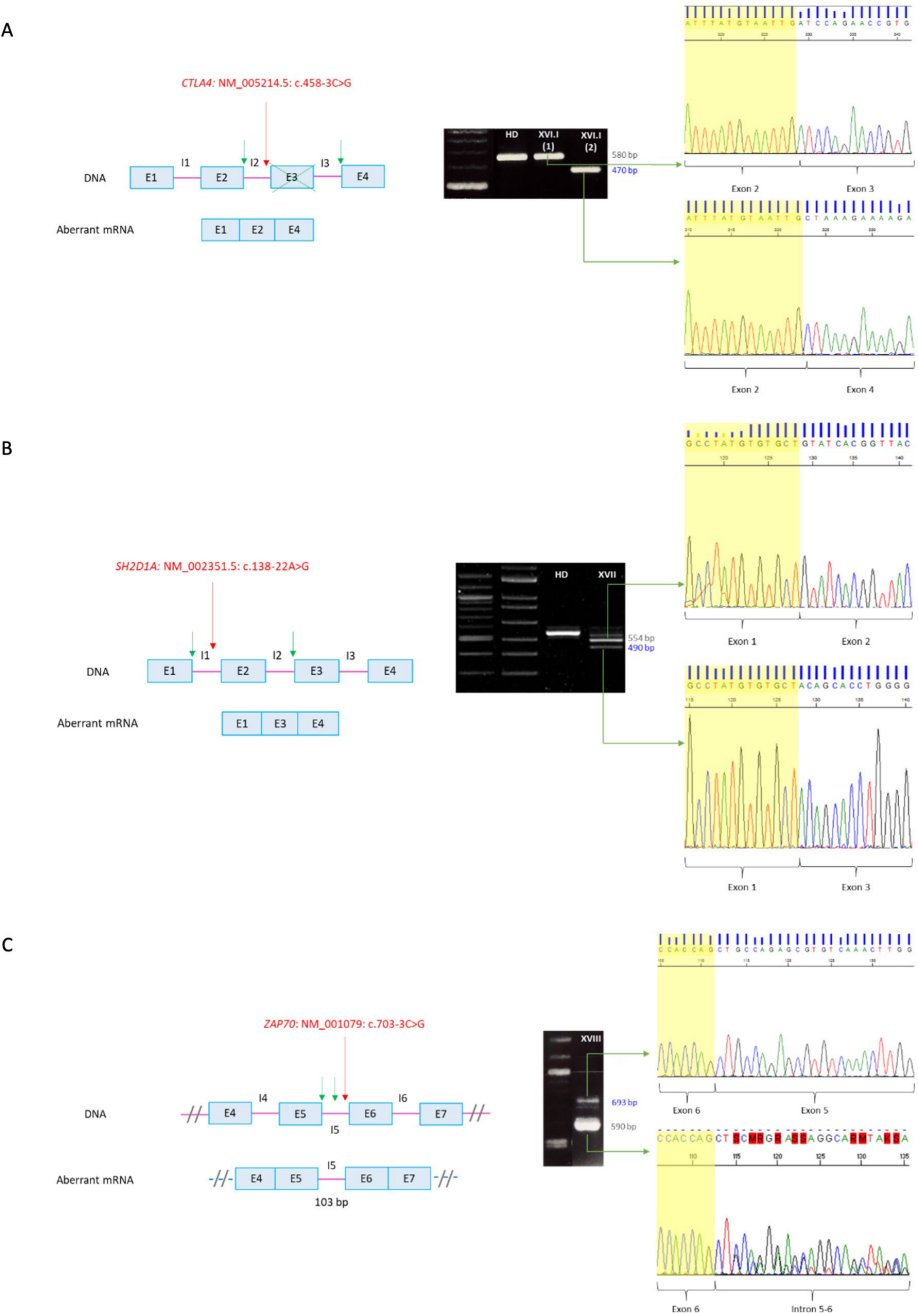
RT-PCR of non-canonical splicing defects. Base pairs corresponding to fragments of the wild type (wt) mRNA are shown in grey and those of the aberrant ones in blue. Red arrows indicate splicing variants positions and green ones point the new splicing sites. E, exon; I, intron; HD, healthy donor; P, proband. **(A)** *CTLA4*: NM_005214.5: c.458-3C>G. Exon 3 skipping on aberrant mRNA. P XVI.I (1) y P XVI.I (2) correspond to positions NM_005214.5: c.104_*9 of the patient belonging to the wt and the aberrant mRNA, respectively. **(B)** *SH2D1A*: NM_002351.5: c.138-22A>G. Exon 2 skipping on aberrant mRNA. The upper band corresponds to the combination of the wild-type mRNA with the aberrant one. **(C)** *ZAP70*: NM_001079: c.703-3C>G. Intron 5 retention on aberrant mRNA. The sequence corresponds to the antisense strand. A double sequence is observed due to contamination of the aberrant mRNA band with the wild-type one. Electrophoresis A and C correspond to the reamplification of two fragments representing the wild type and the aberrant mRNA, obtained from the amplification of both full-length mRNA, whereas electrophoresis B corresponds to the amplification of the full-lenght mRNA, from 5´UTR to 3´UTR.

In patient XVI.I (*CTLA4*: NM_005214.5: c.458-3C>G) two products were obtained in the RT-PCR, which includes the wild type cDNA fragment and a shorter band with an exon 3 skipping leading to a frameshift and a premature stop codon [p.(Asp153AlafsTer23)] (**Figure 3A**). Flow cytometry analysis further revealed 50% of protein expression compared to a healthy donor, which is consistent with the mechanism of haploinsufficiency described for the disease.

RT-PCR of patient XVII (*SH2D1A*: NM_002351.5: c.138-22A>G) yielded two cDNA fragments corresponding to the wild type mRNA in the upper band, whereas de smaller fragment showed an exon 2 skipping leading to a frameshift and a premature stop codon [p.(Tyr47GlnfsTer13)] (**Figure 3B**). The absence of SAP expression was also verified by flow cytometry.

Since patient XVIII had undergone a hematopoietic stem cell transplantation at the time of the study, validation of the variant *ZAP70*: NM_001079: c.703-3C>G was performed on a sample from a healthy heterozygous carrier. The RT-PCR yielded two cDNA fragments (**Figure 3C**). The larger one corresponded to the wild type mRNA, whereas the shorter one showed an intron 5 retention. The resulting aberrant mRNA introduced 103 nucleotides leading to a frameshift and a premature stop codon at the protein level [p.(Leu235ValfsTer95)].

There were two exceptions in which it was not necessary to carry out the validation of the variants and were directly classified as disease causing. In patient XIX (*SERPING1*: NM_000062.3: c.550 + 5G>C), RT-PCR was not required as the variant had previously been reported in multiple affected individuals (13). Besides, low protein levels and impaired functional assay supported the molecular defect and the Alamut software (Interactive BioSoftware, Rouen) indicated that four predictors forecast the loss of the canonical splicing sequence (**Supplementary Figure 1**). In patient XX (*BTK*: NM_000061.3: c.895-11C>A), confirmation of the splicing defect was not needed attending to its functional demonstration in the medical literature, which show exon 11 skipping by RT-PCR and deficient BTK expression by flow cytometry (14).

**Figure 4:**
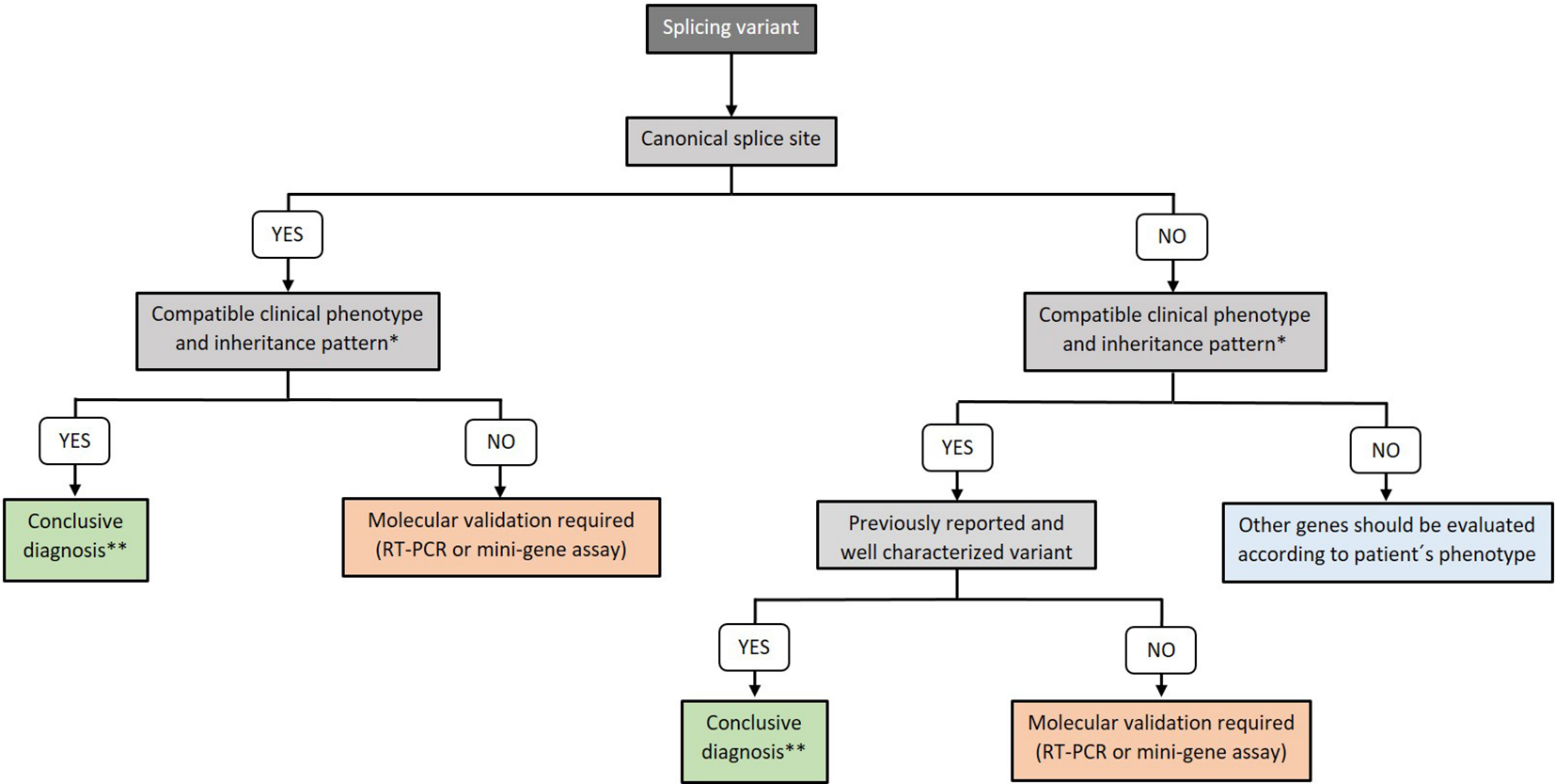
Algorithm for splicing defects molecular confirmation. *: functional and molecular validations are required for GUS since there is no information about the phenotype. **: RT-PCR is recommended for genes with pseudogenes.

Based in our results and experience and attending to the ACMG criteria, we propose an algorithm to determine whether molecular validation is needed to demonstrate the splicing defect or if splicing variant can be directly considered as disease causing (**Figure 4**).

## 4 Discussion

We identified 21 splicing disease-causing variants among 23 patients out of a cohort of 187 patients with IEI confirmed genetic diagnosis, revealing that 12.3% of the IEI were caused by an aberrant splicing process. Splicing variants in IEI cohorts have not been fully studied, and consequently there is not much information about them in the medical literature. However, it is known that specific genes show higher proportions of splicing variants than others, as missense and nonsense variants are the most common changes described in IEI patients. For example, in patients with APDS2, mostly of the PIK3R1 LOF mutations affect splicing and generate an in-frame skipping of exon 11, which leads to the deletion of amino acids 434–475 (15). In the ATM gene, mutations resulting in defective splicing constitute about 48% in patients with ataxia-telangiectasia (16). In other IEI genes, the proportion of splicing defects is significantly lower, such as *TNFAIP3* (7.7%), *BTK* (0.09%) and *CTLA4* (0.08%). In addition, there are genes in which splicing variants have never been reported, including gain of function phenotypes in *STAT1* and *STAT3* (all data from HGMD^®^ Professional database).

Apart from IEI, there are also works on concrete genes that show a significantly higher proportion of splicing variants. For example, in neurofibromatosis type 1 patients, splicing mutations in *NF1* represents the 40% of the amount of causative variants (17), whereas in hereditary breast/ovarian cancer, splicing aberrations in *BRCA1/2* are about 49% (18).

In our cohort, most of the detected variants affect +1/+2, -1/-2 positions at the 5′ donor and the 3′ acceptor sites, respectively. This observation is consistent with the literature, regarding the critical role of these positions in the splicing process (3). Additionally, since the localization of the variant in a canonical splicing position is a very strong criterion for pathogenicity according to ACMG guidelines (11), it is not surprising that these variants are easier to interpret and more frequently reported in the medical literature. Regarding to this, if the splicing variant is located at a canonical position, further validations are only necessary if the clinical phenotype and/or the inheritance pattern are not compatible. In addition, for variants found in a GUS, functional and molecular validations are required since there is no information about the phenotype and the mechanism of the disease, thus ACMG criteria cannot be applied. The validation of variants *IRF4*: NM_002460.4: c. 1213-2A>G and *IRF9*: NM_006084.5: c.577 + 1G>T by RT-PCR, along with other experiments, enabled the first description of IRF4 and IRF9 deficiencies by our group in 2018 and 2019, respectively (19, 20).

Splicing variants often produce a frameshift leading to an early stop codon that results in haploinsufficiency. However, there may be scenarios in which genetic interpretation of the data may be challenging. In our cohort, the RT-PCR of patient XIII revealed that the variant *CARD11*: NM_032415.7: c.358 + 2T>A probably generated a dominant negative effect, as exon 4 skipping was in-frame. Since the mRNA only loses one exon out of 25 and does not produce a premature stop codon, the synthesis of an aberrant protein leading to a dominant-negative mechanism is more likely to occur than the absence of protein expression. A similar IEI case has been reported in the literature by Khourieh et al. (21). The variant *STAT3*: NM_139276.3: c.1282-89C>T in a patient with hiper-IgE syndrome generated an in-frame insertion of 51 nucleotides between exons 14 and 15 that led to an aberrant protein with a dominant negative effect. These challenges in data interpretation also occur in other areas. Damianov and Black (22) reported that Fox proteins produce a different isoform due to an in-frame skipping of RNA-binding domain (RRM) exon. These isoforms act in a dominant negative manner to repress Fox activity. In addition, Nornes et al. (23) showed that splicing defects in the region between exons 6 and 8 of *PSEN1* lead to a truncated protein with a dominant negative effect.

The number of non-canonical splicing variants reported in the medical literature is significantly lower than the number of the canonical-ones. This can be due, in part, to the fact that splicing aberrations are more probable in canonical positions. In addition, misdiagnosis of non-canonical variants may be due to difficulties on data interpretation and the requirement of additional molecular validation. For non-canonical variants, a direct pathogenic effect may be assigned if the variant has previously been reported and the detailed characterization is compatible with the clinical phenotype and inheritance pattern. However, the use of NGS reveals a considerable number of splicing variants outside canonical positions that have not been described in the medical literature and are absent from population databases. In such cases, the clinical phenotype of the patient is of paramount importance, and inheritance pattern must support the presence of the disease. While *in silico* predictors may provide useful insights, molecular validation is essential in cases of high suspicion and will be required to establish a conclusive diagnosis. It is also crucial that the molecular mechanism is compatible. Since it is less common for such variants to result in a gain of function, they are less often observed in disease groups where this mechanism is prevalent. For example, in our cohort, which includes 26 patients with autoinflammatory diseases, frequently associated with gain of function mechanisms, splicing variants were detected in only one patient with ADA2 deficiency, which involves a loss of function mechanism. This represents 3.8% of the variants detected in autoinflammatory disorders, compared to 12.3% observed in the total cohort of IEI patients.

Splicing variants outside canonical positions are not only found near the intron-exon boundaries but also within exons and even in the middle of an intron. In IEI there are many examples of sporadic exonic variants that produce splicing defects. Gallego-Bustos et al. (24) reported the synonymous mutation *IL7R*: NM_002185.5: c.333T>A p.(Val111=), which was found to generate an aberrant mRNA that lacked the last 49 nucleotides of exon 3. The variant *GATA2*: NM_032638.5: c.351C>G p.(Thr117=) reported by Wehr et al. (25) generated an aberrantly spliced transcript by the activation of a cryptic splice donor site removing 136 nucleotides. Additionally, some recurrent mutations have been reported in specific genes. For example, in patients with chronic granulomatous disease (CGD), the variants *CYBB*: NM_000397.4: c.252G>A,T p.(Ala84=) are relatively frequent and lead to exon 3 skipping (26). RT-PCR represents a useful technical approach to reveal the consequences of the defect and correctly classify the variant.

It is important to take into account that deep-intronic variants are not detected by standard sequencing techniques, as these typically sequence exons and adjacent intronic regions. The analysis by RT-PCR for the presence of such mutations should be considered when the clinical suspicion is high and potentially pathogenic variants have not been identified in the coding regions or exon-intron boundaries. Whole genome sequencing (WGS) can be also a helpful tool for this purpose, although the mutations identified on genomic level should be confirmed with functional RNA testing (3). Certain genes are known to have deep-intronic recurrent variants. For example, in patients with GATA2 deficiency, the recurrent mutation *GATA2*: NM_032638.5: c.1017 + 572C>T represent the 25% in some cohorts (27). However, sporadic deep-intronic mutations may occur in any gene. For example, Tateishi et al. (28) reported the variant *BTK*: NM_000061.3: c.840-272G>T in a patient with X-linked agammaglobulinemia (XLA). RT-PCR revealed that the main cDNA fragment had a 122 nucleotides insertion between exons 9 and 10. In addition, Maroilley et al. (29) reported that *ATM*: NM_000051.3: c.1803-270T>G resulted in an aberrant splicing due to the inclusion of 56 intronic nucleotides between exons 11 and 12. RT-PCR also represents a useful approach to achieve a conclusive diagnosis in these clinical scenarios.

Another clinical setting that supports the use of RT-PCR is the confirmation of genomic variants detected by conventional PCR or NGS in genes with highly homologous pseudogenes. As 90% of pseudogenes are not transcribed (10), the detection of the variant in mRNA confirms its existence within the gene sequence. In IEI, this technique may be useful for the study of certain genes in which the presence of pseudogenes is well known. This is the case of *NCF1*, which has two adjacent highly conserved pseudogenes (30), *SBDS* (31) or *DCLRE1C* (32), both with one high homologous pseudogen.

While RT-PCR has numerous benefits, this approach has also some limitations. On one hand, if the splicing defect involves an intron retention, mRNA size can overcome enzyme processivity and, consequently, the cDNA will not be properly amplified. On the other hand, in some cases a protective mechanism, known as nonsense-mediated decay (NMD), leads to mRNA degradation and amplification by RT-PCR is not possible. Alternatively, quantitative PCR (qPCR) may be highly useful, as it allows the quantification of wt mRNA levels. Consistent with this view, Colobran et al. (33) performed qPCR to demonstrate NMD produced by the variant *SERPING1*: NM_000062.3: c.685 + 2T>A, showing that levels of the wild type mRNA were about 50% of those of the healthy donors.

In conclusion, we propose an algorithm to determine when splicing variants should be validated and how RT-PCR can be a useful assay to be performed in genetic diagnosis routine laboratories for those situations in which interpretation of the results is not obvious. WGS can be an important tool for the detection of deep-intronic variants that may lead to an aberrant splicing, although molecular validation will be necessary in positive cases. Further investigation about splicing defects and the description of new patients will extend our knowledge in IEI genes and in human genetic pathology.

## Data Availability

The datasets generated during the current study are not publicly available due to concerns regarding participant/patient anonymity, but are available from the corresponding author on reasonable request.
