# Supplementary material for: Molecular assessment of splicing variants in a cohort of patients with inborn errors of immunity: methodological approach and interpretation remarks
Source: Front Immunol. 2025 Jan 29;15:1499415. doi: 10.3389/fimmu.2024.1499415 (PMC11814461; doi:10.3389/fimmu.2024.1499415)

Supplementary Material

# Supplementary Figures and Tables

# 1.1. Supplementary Tables

| Variant | First amplification primers* | Re-amplification primers** |
| --- | --- | --- |
| *SBDS*: NM_016038.4: c. 258+2T>C | 5´ CTGCCTGAAGCTAGTGAGTC 3´  5´ CGATGAGACAGAGTACGGAAC 3´ | 5´ GCGTGGAAAAAGACCTCGATG 3´  5´ CATACGATTTCTAACTGTTGGC 3´ |
| *CARD11*: NM_032415.7: c.358+2T>A | - | 5´ TCAAGGGACGACAAGATCCA 3´  5´ TTCATTCTCCCGCTCCAGTT 3´ |
| *IRF4*: NM_002460.4: c. 1213-2A>G | 5´ CCTCAGCTCCGAGTCCAG 3´  5´ CCTGTAATCCCAGCTACCAGG 3´ | 5´ CACACAGCAGTTCTTGTCAGAG 3´  5´ CCTGTAATCCCAGCTACCAGG 3´ |
| *IRF9*: NM_006084.5: c.577+1G>T | 5´ GAGGAGAACTGAAACTTAGG 3´  5´ GAGGACAGGTCAATCGTGTG 3´ | 5´ CAGGAATCGTCTCTGGCCA 3´  5´ GACCAAGTCTCTGCAGAAGT 3´ |
| *CTLA4:* NM_005214.5: c.458-3C>G | 5´ GAAGACCTGAACACCGCTCC 3´  5´ CCCATCGAACTGGAGCTTCC 3´ | 5´ GCAAAGCAATGCACGTGG 3´  5´ ATAATGGTTTCTCAATTGATGGG 3´ |
| *SH2D1A*: NM_002351.5: c.138-22A>G | 5´ TGCCCAAGAGTCCACCAG 3´  5´ TGACGACATGCTAGATGCTTC 3´ | - |
| *ZAP70*: NM_001079: c.703-3C>G | 5´ GCATTCAGAACCGGCTCTCC 3´  5´ AGCCAGACTGCAGGACCT 3´ | 5´ AGTTCTACTCGCGCGACC 3´  5´ CTGAGGATGAGTCAACGTGG 3´ |

**Supplementary Table 1**. Primer sequences for RT-PCR. *PCR amplification of the cDNA corresponding to the full-length messenger using a forward primer that hybridizes in the 5’ UTR and a reverse primer that hybridizes in the 3’ UTR. **Nested amplification of a smaller fragment using primers complementary to exon-exon junctions specific to the transcript of interest.

# Supplementary Figures

**Supplementary Figure 1**. Prediction of the impact of the variant *SERPING1*: NM_000062.3: c.550+5G>C on splicing by the Alamut software.


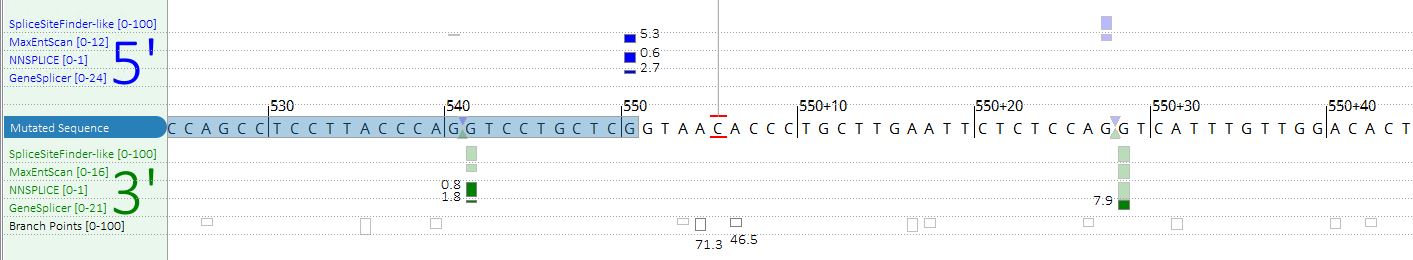

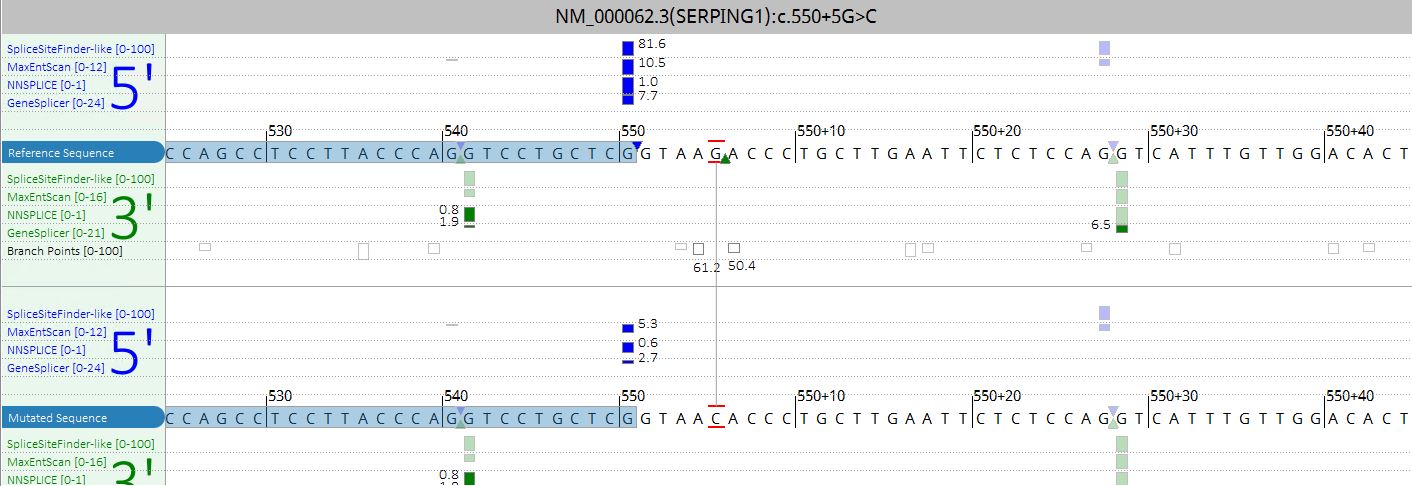

Supplement: Supplementary file 1 [file Table1.docx]
